# Endoplasmic Reticulum Chaperon Tauroursodeoxycholic Acid Attenuates Aldosterone-Infused Renal Injury

**DOI:** 10.1155/2016/4387031

**Published:** 2016-09-19

**Authors:** Honglei Guo, Hongmei Li, Lilu Ling, Yong Gu, Wei Ding

**Affiliations:** ^1^Division of Nephrology, The Fifth People's Hospital of Shanghai, Fudan University, Shanghai, China; ^2^Division of Nephrology, Shanghai Ninth People's Hospital, School of Medicine, Shanghai Jiaotong University, 639 Zhizaoju Road, Shanghai 200011, China

## Abstract

Aldosterone (Aldo) is critically involved in the development of renal injury via the production of reactive oxygen species and inflammation. Endoplasmic reticulum (ER) stress is also evoked in Aldo-induced renal injury. In the present study, we investigated the role of ER stress in inflammation-mediated renal injury in Aldo-infused mice. C57BL/6J mice were randomized to receive treatment for 4 weeks as follows: vehicle infusion, Aldo infusion, vehicle infusion plus tauroursodeoxycholic acid (TUDCA), and Aldo infusion plus TUDCA. The effect of TUDCA on the Aldo-infused inflammatory response and renal injury was investigated using periodic acid-Schiff staining, real-time PCR, Western blot, and ELISA. We demonstrate that Aldo leads to impaired renal function and inhibition of ER stress via TUDCA attenuates renal fibrosis. This was indicated by decreased collagen I, collagen IV, fibronectin, and TGF-*β* expression, as well as the downregulation of the expression of Nlrp3 inflammasome markers, Nlrp3, ASC, IL-1*β*, and IL-18. This paper presents an important role for ER stress on the renal inflammatory response to Aldo. Additionally, the inhibition of ER stress by TUDCA negatively regulates the levels of these inflammatory molecules in the context of Aldo.

## 1. Introduction

The chronic administration of aldosterone (Aldo) leads to the production of reactive oxygen species (ROS) and causes endothelial dysfunction, disruption of the glomerular filtration barrier, proteinuria, and tubular damage and regeneration, leading to the progression of chronic kidney disease [1–5]. Aldo exhibits these classic actions by combining with the mineralocorticoid receptor (MR), a member of the nuclear receptor family of proteins. Additionally, an MR antagonist can attenuate renal injury via reducing the level of ROS generation [6]. Recently, ROS was identified as an initiator and major contributor to ER stress [7]. Reports have shown that endoplasmic reticulum (ER) stress facilitates fibrotic remodeling through the promotion of inflammatory responses [8]. Excessive ER stress may result in fibrosis through activating CHOP-mediated apoptosis and a subsequent response of inflammatory and profibrotic cytokines [9]. ER stress can elicit an inflammatory response through the induction of the ASC and Nlrp3 inflammasome [10, 11]. This in turn promotes the maturation and secretion of proinflammatory cytokines, including IL-1*β* and IL-18, to initiate innate immune defenses and subsequently results in cellular injury [12]. The Nlrp3 inflammasome is a crucial event in the progression of kidney disease [13–16], including unilateral ureteral obstruction (UUO) [13], ischemia/reperfusion injury [17, 18], and proteinuric animal model [19]. In those models, Nlrp3^−/−^ mice were remarkably resistant to renal injury, possibly via the inhibition of the inflammatory response. Aldo-driven renal injury also consists of inflammatory components involving IL-1*β* and IL-6 upregulation. These effects are attenuated by eplerenone, supporting the protective effect of an Aldo blockade in renal disease via inhibiting inflammatory cytokines [20].

Our previous studies have demonstrated that TUDCA could ameliorate renal injury and ER stress-mediated uremic cardiomyopathy [10]. Chiang and coworkers also demonstrated the inhibition of ER stress by TUDCA, an ER stress inhibitor, protected against UUO-induced renal fibrosis [21]. Collectively, these studies indicate that inhibition of ER stress may be one of the possible therapeutic targets against renal fibrosis and injury. However, the molecular mechanisms leading to the renal inflammatory changes that occur in response to ER stress have not been reported in detail. The present study explores the potential role of ER stress in Aldo-infused renal inflammation and fibrosis.

## 2. Materials and Methods

### 2.1. Animal Models

All experiments were performed in accordance with the Fudan Medical University Guide for Laboratory Animals. Eight-week-old C57BL/6J mice weighting between 25 and 30 g were purchased from the Institute of Animal Care at Fudan University and underwent a right uninephrectomy under anesthesia with sodium pentobarbital (50 mg/kg, IP). After two weeks of recovery, all mice were given drinking water containing 1% NaCl and randomly treated with one of the following for four weeks: group 1, Sham+V (0.5% ethanol subcutaneously, saline vehicle i.p., *n* = 6); group 2, Sham+TUDCA (0.5% ethanol subcutaneously, 250 mg/kg/d of TUDCA i.p., Sigma-Aldrich, USA, *n* = 6); group 3, Aldo+V (Aldo 0.75 *μ*g/h subcutaneously, Sigma-Aldrich, USA, saline vehicle i.p., *n* = 6); and group 4, Aldo+TUDCA (Aldo 0.75 *μ*g/h subcutaneously, 250 mg/kg/d of TUDCA i.p., *n* = 6) [10]. At the end of the experiment, the mice were anesthetized and the body and kidney weight were measured. Twenty-four-hour urine samples were collected after a 24 h acclimatization period in the metabolic cages. Urinary protein excretion was determined using enzyme-linked immunosorbent assay (ELISA) kits (Exocell). Additionally, the plasma was centrifuged for testing creatinine, urea nitrogen, IL-18, and IL-1*β*. Kidney samples were immediately frozen in liquid nitrogen and stored at −80°C.

### 2.2. Kidney Histopathological Analysis

Kidney tissues were fixed in 4% paraformaldehyde, embedded in paraffin, and sliced at a thickness of 3 *μ*m per section. They were stained with periodic acid-Schiff (PAS) according to a standard protocol [22]. The severity of glomerular injury from each mice kidney section was assessed using light microscopy. Then, the sections were rated for glomerular proliferative lesions on a scale from 0 to 4 as follows: 0 equated to no proliferation, whereas 1+, 2+, 3+, and 4+ corresponded to 1%–25%, 26%–50%, 51%–75%, and 76%–100% of segmental lesion per glomeruli, respectively.

### 2.3. Detection of ROS

We detected renal thiobarbituric acid reactive substances (TBARS) using commercial kits (Cayman Chemical Company). Moreover, we measured serum malondialdehyde (MDA) and 8-OHdG using commercial kits (Jiancheng Bioengineering Research Institute) according to the manufacturer's protocol.

### 2.4. Quantitative Real-Time PCR

The total RNA was extracted from the kidneys using a kit (Fermentas, Glen Burnie, MD, USA) according to the manufacturer's protocol. Real-time PCR amplification was performed using the SYBR Green master mix (ABI, USA) and the Prism 7300 real-time PCR detection system (Applied Biosystems). Oligonucleotide sequences were provided by Invitrogen and the primer pairs were presented in Table 1 [10]. The expression of mRNA levels was normalized by subtracting the corresponding GAPDH as a control and calculated by using the comparative cycle threshold method.

### 2.5. Western Blotting and ELISA

The kidney tissues were homogenized and the supernatant was collected after centrifugation at 12,000 ×g at 4°C for 20 min [23]. We separated the lysates on 10% polyacrylamide gels before immunoblotting using anti-Nlrp3 (AdipoGen company, San Diego, CA), anti-ASC (AdipoGen company, San Diego, CA), anti-CHOP (Cell Signaling Technology, USA), anti-caspase-12 (Cell Signaling Technology, USA), anti-IL-1*β* (Affinity Biosciences, USA), and anti-IL-18 (Affinity Biosciences, USA) antibodies at a dilution of 1 : 500. The expression levels of CHOP, caspase-12, ASC, Nlrp3, IL-1*β*, and IL-18 were analyzed using an ECL advance system (Amersham, Little Chalfont, UK). The relative protein expression levels were determined by normalization to *β*-actin. Serum IL-1*β* and IL-18 were measured with ELISA kits (RayBiotech, Norcross, GA) according to the manufacturer's instruction.

### 2.6. Statistical Analysis

Results were expressed as means ± the standard error of the mean (SEM). A one-way ANOVA was used to compare mean values, and a value of *p* < 0.05 was determined to be statistically significant.

## 3. Results 

### 3.1. Effects of TUDCA on Renal Function in Aldo-Infused Mice

**Figure 1:**
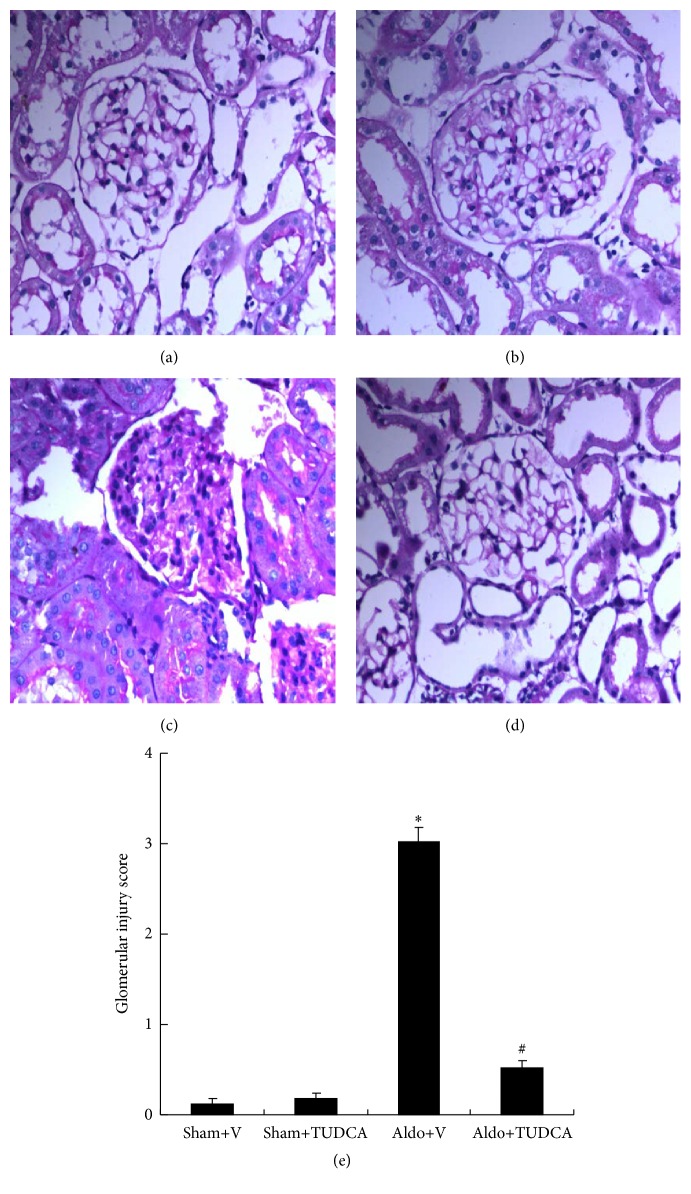
Physiologic parameters of the mice at the end of week 4. Representative photomicrographs (magnification: 400x) of PAS-stained renal injury. (a) Sham+V group; (b) Sham+TUDCA group; (c) Aldo+V group; (d) Aldo+TUDCA group; (e) Glomerular Injury Score. ^*∗*^ *p* < 0.05, Sham+V group versus Aldo+V group, ^#^ *p* < 0.05, Aldo+V group versus Aldo+TUDCA group.

Renal damage was assessed by PAS staining, serum creatinine, albumin/creatinine, and BUN. As shown in Figure 1, Aldo-infused mice showed markedly expanded mesangial regions and glomerulosclerosis (3.02 ± 0.16) compared to the Sham+V group (Glomerular Injury Score: 0.13 ± 0.05). However, treatment with TUDCA significantly mitigated renal injury and reduced the Glomerular Injury Score (0.52 ± 0.09). Consistent with this finding for PAS staining, levels of BUN and albumin/creatinine were also significantly increased in Aldo+V mice (65.4 ± 3.96 mg/dL and 102.5 ± 12.77, resp.) compared to Sham+V mice (24.5 ± 0.83 mg/dL and 25.5 ± 3.04, resp.). Levels of BUN and albumin/creatinine were markedly decreased in Aldo+TUDCA mice (37.4 ± 1.97 mg/dL and 44.8 ± 5.26, resp.) relative to Aldo+V mice. The creatinine concentration was higher in Aldo-infused mice (0.30 ± 0.02) compared to Sham+V mice (0.21 ± 0.01), and treatment with TUDCA decreased the level of creatinine in the Aldo-treated group (0.26 ± 0.01) (Table 2).

### 3.2. Effects of TUDCA on Renal Fibrosis

**Figure 2:**
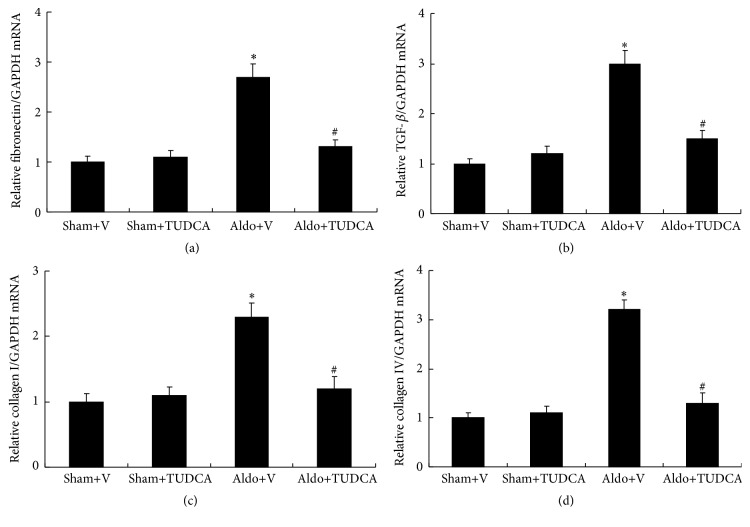
Expression of fibrotic cytokines in the kidney. (a) mRNA expression of fibronectin, (b) TGF-*β*, (c) collagen I, and (d) collagen IV was detected by real-time PCR and normalized to expression of glyceraldehyde 3-phosphate dehydrogenase. Values are mean ± SEM (*n* = 6). ^*∗*^ *p* < 0.05, Sham+V group versus Aldo+V group, ^#^ *p* < 0.05, Aldo+V group versus Aldo+TUDCA group.

Compared with the Sham+V mice, the Aldo+V group demonstrated significantly increased mRNA levels. mRNA levels of fibronectin, transforming growth factor-*β* (TGF-*β*), collagen I, and collagen IV were increased remarkably in Aldo+V group (2.7-fold, 3.0-fold, 2.3-fold, and 3.2-fold, resp.) compared to the Sham+V mice. In addition, TUDCA treatment significantly decreased the mRNA levels of fibronectin, TGF-*β*, collagen I, and collagen IV compared with those of Aldo+V mice (Figure 2).

### 3.3. Effects of TUDCA on Kidney ROS

**Figure 3:**
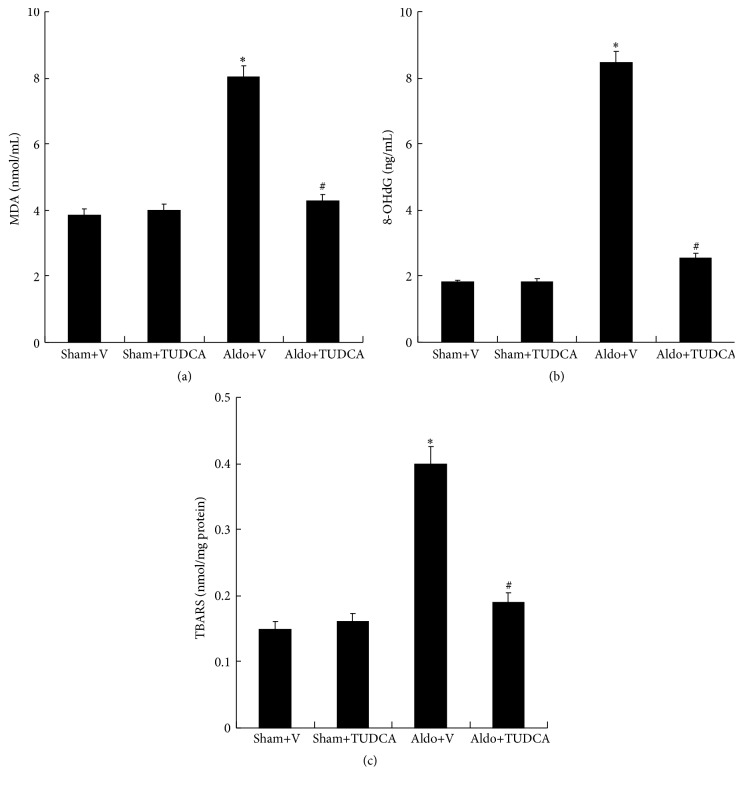
ROS levels in the mouse kidney. (a) MDA, (b) 8-OHdG, and (c) TBARS were detected according to the manufacturer's protocol. Values are mean ± SEM (*n* = 6). ^*∗*^ *p* < 0.05, Sham+V group versus Aldo+V group, ^#^ *p* < 0.05, Aldo+V group versus Aldo+TUDCA group.

Indices of ROS production, including MDA (8.03 ± 0.33), 8-OHdG (8.46 ± 0.32), and TBARS (0.4 ± 0.03), were significantly increased in Aldo+V group compared with the Sham+V group (3.86 ± 0.18, 1.81 ± 0.08, and 0.15 ± 0.01). In contrast, treatment with TUDCA significantly attenuated the Aldo-induced elevated levels of MDA, 8-OHdG, and TBARS (Figure 3).

### 3.4. Effects of TUDCA on the ER Stress-Induced Apoptotic Pathway in Mouse Kidney

**Figure 4:**
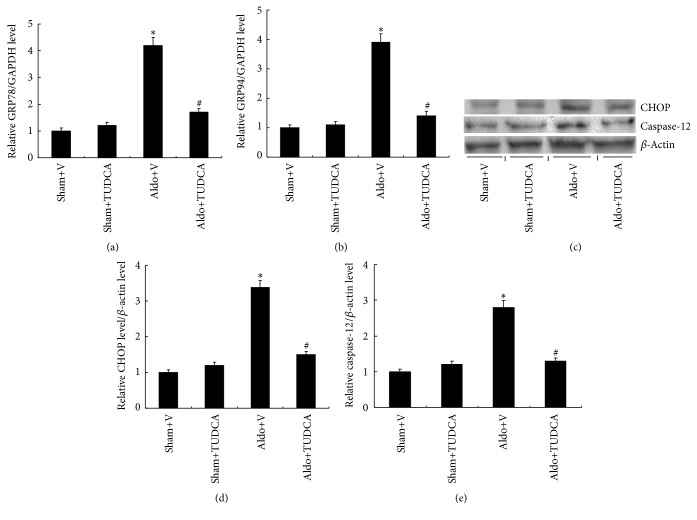
TUDCA ameliorated Aldo-induced ER stress. (a) Relative mRNA expression of GRP78 compared to expression of GAPDH. (b) mRNA expression of GRP94 relative to the expression of GAPDH. (c) Representative Western blots of CHOP and caspase-12. (d) Relative expression of CHOP to expression of *β*-actin. (e) Relative expression of caspase-12 to expression of *β*-actin. Values are mean ± SEM (*n* = 6). ^*∗*^ *p* < 0.05, Sham+V group versus Aldo+V group, ^#^ *p* < 0.05, Aldo+V group versus Aldo+TUDCA group.

GRP 78 and GRP 94 as ER stress markers are critical regulators of ER function. Expression of GRP78 (4.2-fold) and GRP94 (3.9-fold) are increased markedly in mouse kidneys of Aldo+V mice relative to Sham+V mice. However, TUDCA treatment reduced the levels of GRP 78 and GRP 94 significantly (Figure 4). Activation of ER stress can initiate apoptosis via CHOP and caspase-12 pathways. Levels of CHOP (3.4-fold) and caspase-12 (2.8-fold) were increased significantly in the Aldo+V group compared with the Sham+V mice. Moreover, TUDCA treatment reduced the levels of CHOP and caspase-12 remarkably (Figure 4). This demonstrates the important role of CHOP and caspase-12 signaling in Aldo-driven renal injury.

### 3.5. Effects of TUDCA on Nlrp3 Inflammasome Activation in the Kidney

**Figure 5:**
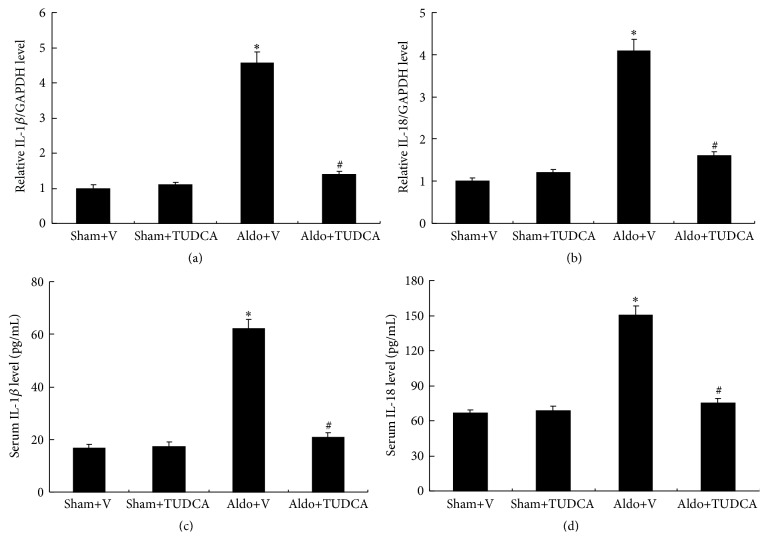
TUDCA decreased IL-1*β* and IL-18 levels in Aldo-infused mice. (a) Relative mRNA expression of IL-1*β* to expression of GAPDH. (b) Relative mRNA expression of IL-18 to the expression of GAPDH. (c) Serum IL-1*β* levels and (d) serum IL-18 levels. Values are mean ± SEM (*n* = 6). ^*∗*^ *p* < 0.05, Sham+V group versus Aldo+V group, ^#^ *p* < 0.05, Aldo+V group versus Aldo+TUDCA group.

**Figure 6:**
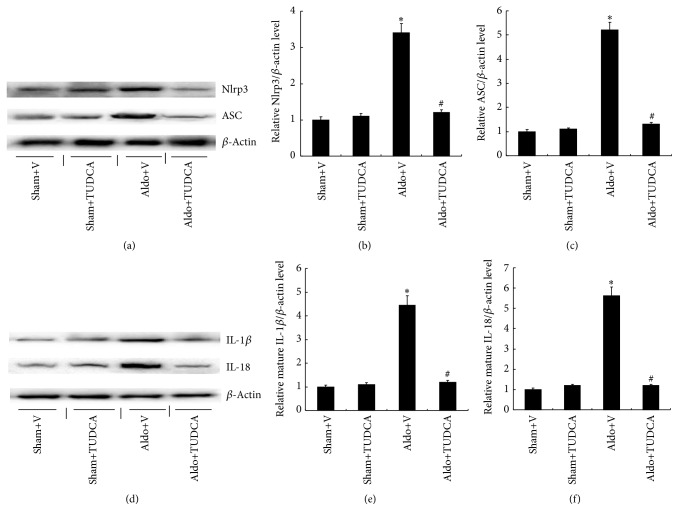
TUDCA attenuated Nlrp3 inflammasome activation in Aldo-infused mice. (a) Representative Western blots of Nlrp3 and ASC. (b) Relative expression of Nlrp3 to the expression of *β*-actin. (c) Relative expression of ASC to the expression of *β*-actin. (d) Representative Western blots of IL-1*β* and IL-18. (e) Relative expression of IL-1*β* to expression of *β*-actin. (f) Relative expression of IL-18 to the expression of *β*-actin. Values are mean ± SEM (*n* = 6). ^*∗*^ *p* < 0.05, Sham+V group versus Aldo+V group, ^#^ *p* < 0.05, Aldo+V group versus Aldo+TUDCA group.

The real-time PCR analysis demonstrated that mRNA expression of Nlrp3 inflammasome-related genes, including IL-1*β* (4.6-fold) and IL-18 (4.1-fold), was significantly increased in the kidneys of Aldo+V mice relative to Sham+V mice. Treatment with TUDCA markedly decreased the levels of IL-1*β* and IL-18 (Figure 5). Similarly, the levels of serum IL-1*β* (62.2 ± 3.5) and IL-18 (150.5 ± 7.6) were significantly increased in Aldo+V mice compared with Sham+V mice (16.8 ± 1.5 and 66.3 ± 3.2, resp.), whereas the levels of IL-1*β* and IL-18 were markedly decreased in Aldo+TUDCA mice. Since activated Nlrp3 and ASC proteins cause the subsequent maturation of proinflammatory cytokines, the protein levels of mature IL-1*β* and IL-18 were measured in the mouse kidneys by Western blot. The protein levels of IL-1*β* (4.5-fold) and IL-18 (5.6-fold) were higher in Aldo+V mice compared to the Sham+V group, while TUDCA decreased both cytokines in Aldo-infused mice. Similarly, Nlrp3 (3.4-fold) and ASC (5.2-fold) protein expression were also increased in the Aldo+V group compared with the Sham+V group. Treatment with TUDCA was able to significantly decrease these protein levels in Aldo+V mice (Figure 6).

## 4. Discussion

This study demonstrated that ROS, ER stress, and renal Nlrp3 inflammasome were increased in Aldo-infused mice. In addition, treatment with TUDCA, an ER stress inhibitor, was shown to prevent Nlrp3 inflammasome activation and its related cytokines. This indicates that TUDCA may ameliorate Aldo-infused renal injury via inhibiting activation of the NLRP3 inflammasome.

ROS overproduction has been correlated with a variety of renal injury models, including diabetic kidney disease, focal segmental glomerulosclerosis, and membranous nephropathy [24]. Several reports have demonstrated that excessive Aldo in animal models was associated with mesangial cell and podocyte injury, due to ROS activation [24, 25]. Previous studies have also shown that ROS overproduction leads to oxidative stress and triggers redox-sensitive cell signaling cascades that elicit an inflammatory response, mitochondrial dysfunction, and fibrogenesis. Moreover, antioxidants and free radical scavengers partially improved proapoptotic outcomes of Aldo [26]. ROS was also identified as a potential trigger of the Nlrp3 inflammasome in podocytes [13]. Therefore, these results indicate that it is likely that ROS is involved in the activation of the inflammasome in Aldo-driven renal injury [13, 27–30]. The present study demonstrated that ROS markers were activated following Aldo-infused renal injury. Oxidative stress is a known inducer of ER stress. Oxidant stress disrupts the ER homeostasis and activates ER stress in the kidneys and is associated with Aldo-driven renal injury. Although the detailed mechanisms between ROS and ER stress in Aldo-induced inflammation remain unknown, these findings provide important insight into the renal injury in response to Aldo.

Increasing evidence has demonstrated that ER stress and Nlrp3 inflammasome activation are important pathogenic factors in multiple kidney diseases [2, 16–18]. Previous studies have also reported that the levels of ER stress protein in human and experimental animal models were significantly upregulated following Aldo treatment [31, 32]. CHOP, an ER stress activation marker, is commonly expressed at low levels and is robustly activated in a wide variety of organs as part of the natural stress response. The present study showed that Aldo exposure substantially increased CHOP expression. However, how ER stress contributes to Aldo-evoked renal injury remains unknown. Previous reports demonstrated that ER-induced renal injury can induce autophagy [7, 31]. However, the precise mechanism by which this occurs was not determined. In addition, activation of autophagy in animal experiments exhibited only a partial resistance to ER stress-induced renal injury, indicating that autophagy was not a unique pathway of fibrosis.

Inflammation appears to be an important destructive process that mediates injury in the kidneys. Moderate tubulointerstitial inflammation and fibrosis have been shown to be mediated through Nlrp3 inflammasome activation as indicated by the elevated mRNA and protein levels of Nlrp3 and ASC [33]. Aldo is able to facilitate the initiation and maintenance of inflammatory cells into the vascular wall to promote smooth muscle cell proliferation and decrease endothelial function. This in turn accelerates the development of atherosclerosis and stimulates the progression of tissue injury [34–36]. Consistent with previous studies, this study also showed that the Nlrp3 inflammasome was present in the Aldo-infused model of renal injury. Similar to previous findings, increased protein levels of Nlrp3 and ASC and the activation of matured IL-18 and IL-1*β* were observed. Taken together, these results suggest that the Nlrp3 inflammasome may contribute to Aldo-induced renal injury. Treatment with TUDCA markedly attenuated kidney disease and decreased Nlrp3 inflammasome activation, indicating that the Nlrp3 inflammasome may be downstream of the ER stress pathway in Aldo-induced kidney disease.

Prolonged ER stress contributed to fibrosis via increased inflammatory responses and the generation of profibrotic cytokines [36]. In addition, activation of ER stress in immune cells induced the production of proinflammatory cytokines. ER stress was also identified to play an important role in inducing inflammation and the release of TGF-*β* in the CHOP-mediated activation of apoptosis [9].

We previously demonstrated that TUDCA significantly ameliorated renal function and uremic cardiomyopathy via inhibiting ER stress pathway [10]. Fang et al. [37] showed that albuminuria induced inflammasome activation via ER stress signaling in renal proximal tubular cells. The present study demonstrated that TUDCA alleviated ER stress response driven by Aldo in renal injury. Treatment with TUDCA decreased ER stress proteins including GRP78, GRP94, CHOP, and caspase-12. In addition, TUDCA alleviated inflammation induced injury via downregulating ASC and NLRP3. Blocking the ER stress pathway can inhibit Aldo-driven production of IL-18 and IL-1*β*. The results above suggest that Aldo may activate the Nlrp3 inflammasome via the ER stress response, indicating the important crosstalk between ER stress pathway and Nlrp3 inflammasome activation in Aldo-induced renal injury. This data provides indirect evidence supporting the pathological role of the Nlrp3 inflammasome induced by Aldo in renal injury. Further* in vitro* studies are necessary to identify the possible involvement of ER stress in Aldo-driven Nlrp3 inflammasome.

In conclusion, the present study presents an important role of ER stress on renal inflammation, responses to Aldo, and ER stress inhibitors in the context of inflammation ameliorated renal fibrosis. The inhibition of ER stress, as well as Nlrp3 and ASC, suggests that redundant inflammatory pathways are involved in Aldo-induced chronic kidney disease. Furthermore, treatment with TUDCA significantly attenuates the Nlrp3 inflammasome, suggesting that the ER stress pathway may mediate Nlrp3 inflammasome activation in Aldo-infused renal injury.

## Figures and Tables

**Table 1:** Oligonucleotides synthesized by Invitrogen (Carlsbad, CA, USA).

	Forward	Reverse
Transforming growth factor-beta	5′-AGCTTTGCAGGGTGGGTATC-3′	5′-CCTTCGGGTGAGACCACAAA-3′
Fibronectin	5′-GCGACGGTATTCTGTAAAGTGG-3′	5′-GGACAGGGCTTTGGCAGTT-3′
Collagen I	5′-AGGGTCATCGTGGCTTCTCT-3′	5′-CAGGCTCTTGAGGGTAGTGT-3′
Collagen IV	5′-ATCGGATACTCCTTCCTCATGC-3′	5′-CCAGGGGAGACTAGGGACTG-3′
GRP78	5′-CTGCTGAGGCGTATTTGGGAAA-3′	5′-TCAATGGTGAGAAGAGACACATCG-3′
GRP94	5′-GTCGTGGAACAACAATTACTCTTG-3′	5′-GCTTCATCATCAGATTCTTCTTTCTC-3′
IL-1*β*	5′-AGCCTTTGTCCTCTGCCAAGT-3′	5′-CCAGAATGTGCCACGGTTTT-3′
IL-18	5′-GGGATGGGAGGAACGCTACTA-3′	5′-ACAGGTTGTACTGGAAAAGCC-3′
Glyceraldehyde 3-phosphate dehydrogenase	5′-TCAGCCGCATCTTCTTTTG-3′	5′-AAATCCGTTGACTCCGACC-3′

**Table 2:** Biological parameters in Aldo-infused mice at week 4.

	Sham+V	Sham+TUDCA	Aldo+V	Aldo+TUDCA
Body weight (g)	27.06 ± 1.25	27.18 ± 1.32	26.54 ± 1.85	26.88 ± 1.98
Kidney weight/body weight ratio (mg/g)	10.04 ± 0.21	10.11 ± 0.28	14.73 ± 0.22^*∗*^	11.15 ± 0.25^#^
Albumin/creatinine (ug/mg)	25.5 ± 3.04	26.4 ± 2.72	102.5 ± 12.77^*∗*^	44.8 ± 5.26^#^
Serum creatinine (mg/dL)	0.21 ± 0.01	0.25 ± 0.01	0.30 ± 0.02	0.26 ± 0.01
BUN (mg/dL)	24.5 ± 0.83	25.1 ± 1.07	65.4 ± 3.96^*∗*^	37.4 ± 1.97^#^

Data are presented as mean ± SEM; (*n* = 6); ^*∗*^ *p* < 0.05, Sham+V versus Aldo+V; ^#^ *p* < 0.05, Aldo+V versus Aldo+TUDCA.
